# High‐Pressure Homogenization of Pomegranate Juice: Impact on Physicochemical, Antioxidant, Antimicrobial, and In Vitro Bioaccessibility Properties

**DOI:** 10.1002/fsn3.4571

**Published:** 2024-11-07

**Authors:** Emre Turan, Rafet Aslantaş, Jale Bilgin, Muhammet Irfan Aksu

**Affiliations:** ^1^ Department of Food Engineering, Faculty of Agriculture Ordu University Ordu Türkiye; ^2^ Department of Horticulture, Faculty of Agriculture Eskişehir Osmangazi University Eskişehir Türkiye; ^3^ Department of Food Engineering, Faculty of Agriculture Atatürk University Erzurum Türkiye

**Keywords:** antioxidant capacity, bioaccessibility, bioactive compounds, high‐pressure homogenization, in vitro gastrointestinal digestion, pomegranate

## Abstract

Pomegranate is one of the most popular fruits worldwide, and it is important to maintain the overall quality and bioaccessibility of freshly squeezed pomegranate juices (PJS). The adverse effects of heat treatment on sensory properties and phytochemicals encourage the use of non‐thermal processes in the juice industry. Hereby, the effects of high‐pressure homogenization (HPH) (50, 100, and 150 MPa) on the physicochemical properties, antimicrobial activity, in vitro bioaccessibility, and antioxidant capacity of freshly‐squeezed PJS from different genotypes were investigated. Instrumental color values (L*, a*, and b*) of PJS generally decreased, while pH and *a*_*w*_ values increased with HPH processing (*p* < 0.05). The antimicrobial activity of PJS was not significantly affected by HPH processing (*p* > 0.05). Sarıcakaya‐1 had higher bioactive constituent content and antioxidant capacity (DPPH‐ARA, ABTS‐ARA, and FRAP) than other genotypes (*p* < 0.05). Total phenolic content (TPC) and antioxidant capacity of PJS were increased by HPH treatment, whereas total anthocyanin content (TAC) and ascorbic acid content (AAC) were decreased (*p* < 0.05). Accordingly, HPH treatment enhanced the release of phenolics and antioxidant capacity in PJS, while largely maintaining the presence of heat‐sensitive compounds. Regardless of genotype and HPH treatment, in vitro gastrointestinal digestion adversely affected TPC, TAC, AAC, and antioxidant capacity of all PJS. However, the 150 MPa HPH treatment of Saricakaya‐1 had the highest values for TPC bioaccessibility and recovered ABTS‐ARA after in vitro gastrointestinal digestion (*p* < 0.05). Considering the overall data, 150 MPa HPH treatment can be recommended as an innovative approach to obtain minimally processed PJS to meet new trends in consumer demand.

## Graphical abstract

The present study showed that HPH treatment increased the release of phenolics and antioxidant capacity in pomegranate juices (PJS), while largely maintaining the presence of heat‐sensitive compounds. Moreover, HPH processing did not adversely affect the bioaccessibility of PJS; on the contrary, 150 MPa treatment contributed positively. These results underline the promising potential of HPH for the production of minimally processed juices that meet the new trends in consumer demand.

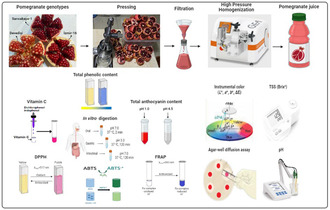

## 1 Introduction

Pomegranate (*Punica granatum* L.) is one of the most popular fruits known for its unique color, flavor, and health‐promoting properties (Benjamin and Gamrasni 2020; Yuan et al. 2022). Each part of the pomegranate (i.e., seeds, arils, and peel) is rich in bioactive compounds, and especially pomegranate juices (PJS) are an important source of organic acids, vitamins, sugars, minerals, and polyphenol compounds (Putnik et al. 2019; Topalović et al. 2021). Pomegranate has antioxidant, antimicrobial, anti‐cancer, and anti‐inflammatory properties, as well as preventive and therapeutic effects in various chronic diseases (Cheng et al. 2023). Polyphenols (anthocyanins, ellagitannins, phenolic acids, flavanols, flavonols, and proanthocyanidins), vitamin C, and minerals are the main components responsible for the bioactive properties and health benefits of pomegranate (Benjamin and Gamrasni 2020; Kalaycioglu and Erim 2017). On the other hand, the biological effect of phytochemicals on health depends on the amount of consumption and bioaccessibility. Bioavailability encompasses the processes of release from the food matrix, gastrointestinal digestion, absorption, and metabolism (He et al. 2016). Due to the complexity, high cost, and ethical concerns of in vivo studies, in vitro protocols are preferred when evaluating bioavailability because they offer advantages such as practicality, reproducibility, and low cost (He et al. 2016; Quan et al. 2020). The chemical composition, physicochemical properties, antioxidant potential, and bioavailability of bioactive constituents of pomegranate are significantly influenced by cultivars, climatic conditions, ripening, and processing methods (Çam, Hışıl, and Durmaz 2009; Putnik et al. 2019; Mihaylova et al. 2021).

Despite the increasing consumer demand for freshly squeezed juices, the susceptibility of unprocessed these products to microbial and enzymatic degradation results in limited shelf life (Varela‐Santos et al. 2012). Thermal processes (60°C–90°C, < 1 min) are widely used in the food industry to extend the shelf life of fruit juice by reducing the microbial load and inactivating endogenous enzymes such as polyphenol oxidase. However, undesirable changes in sensory properties and inevitably losses in heat‐sensitive phytochemicals (anthocyanins and ascorbic acid, etc.) depending on the applied temperature and time are important shortcomings of heat treatment (Varela‐Santos et al. 2012; Benjamin and Gamrasni 2020). Moreover, an undesirable color loss occurs after heat treatment, especially in fruit juices containing anthocyanins (Varela‐Santos et al. 2012; Yuan et al. 2022). To overcome these drawbacks, there is a great interest in alternative non‐thermal technologies that provide the benefits of heat treatment in food products.

Nowadays, the consumption of natural and minimally processed products has gained popularity. This encourages all stakeholders in the juice sector to implement innovative non‐thermal technologies such as ultrasound, high‐pressure processing (HPP), pulsed electric field, ultraviolet irradiation, high‐pressure carbon dioxide, and cold plasma (Marszałek et al. 2017; Putnik et al. 2019). Among these clean‐label approaches, high‐pressure homogenization (HPH) is a non‐thermal process in the food industry to inactivate spoilage‐causing or pathogenic microorganisms, prepare emulsions, reduce particle size, and improve the rheological properties of foods (Yong, Song, and Choo 2021). This innovative approach provides food safety and better maintenance of nutrient content and bioactive properties without adversely affecting the sensory properties of liquid products and is considered a promising alternative to thermal processes (Marszałek et al. 2017; Yong, Song, and Choo 2021; Lima and Rosenthal 2023). Preservation of heat‐sensitive bioactive compounds by HPH treatment results in more efficient extraction and improved bioaccessibility of these compounds in some cases (Lima and Rosenthal 2023). HPH treatment can improve sensory properties by increasing the physical stability of the juice, reducing/preventing pulp precipitation during storage, and increasing the consistency of the juice. It can also contribute to reducing the use of additives and hydrocolloids in the juice industry (Augusto, Tribst, and Cristianini 2018).

The principle of HPH is that the fluid is forced through a narrow gap under pressure, increasing its velocity, and causing particle breakup by creating cavitation, shear stress, collision, and turbulence following the pressure drop (Augusto, Tribst, and Cristianini 2018; Yong, Song, and Choo 2021). HPH is also a green technology because the process not only requires solvent use and carbon dioxide emissions but also has a short processing time and low energy consumption. On the other hand, batch systems such as HPP offer a relatively low processing capacity. In contrast, HPH is a continuous flow process that allows homogenization and pasteurization, or in some cases sterilization of liquids in a single step (Yong, Song, and Choo 2021). In this context, HPH technology has promising potential for food applications.

The objectives of this study were: (1) to evaluate the effects of HPH technology on the physicochemical properties and antimicrobial activity of PJS from different Turkish genotypes, (2) to determine the bioaccessibility of bioactive components and the change in antioxidant capacity after in vitro simulated digestion in HPH‐treated PJS.

## 2 Material and Methods

### 2.1 Chemical and Reagents

DPPH (2,2‐diphenyl‐1‐picryl‐hydrazyl), ABTS (2,2′‐Azino‐bis(3‐ethylbenzothiazoline‐6‐sulfonic acid) diammonium salt), potassium persulfate, Trolox ((±)‐6‐Hydroxy‐2,5,7,8 tetramethylchromane‐2‐carboxylic acid), TPTZ (2,4,6‐tripyridyl‐striazine), ascorbic acid, 2,6‐dichlorophenol indophenol, bile (B8631), pancreatin (P7545), pepsin (P7012) were purchased from Sigma‐Aldrich (Steinheim, Germany). Brain Heart Infusion (BHI) broth, Sabouraud Dextrose (SD) broth, Mueller‐Hinton Agar (MHA), Potato Dextrose Agar (PDA), Folin–Ciocalteu reagent, sodium carbonate, gallic acid, ethanol, methanol, potassium chloride, sodium acetate, xylene, hydrochloric acid (HCl), iron (III) chloride hexahydrate (FeCl_3·_6H_2_O), potassium phosphate, sodium bicarbonate, sodium chloride, magnesium chloride, ammonium carbonate, sodium hydroxide (NaOH) and calcium chloride (CaCl_2_) were used from Merck (Darmstadt, Germany). Gentamicin and Amphotericin B antibiotics were procured from the manufacturer (Bioanalyse, Türkiye).

### 2.2 Preparation of PJS and HPH Processing

**FIGURE 1:**
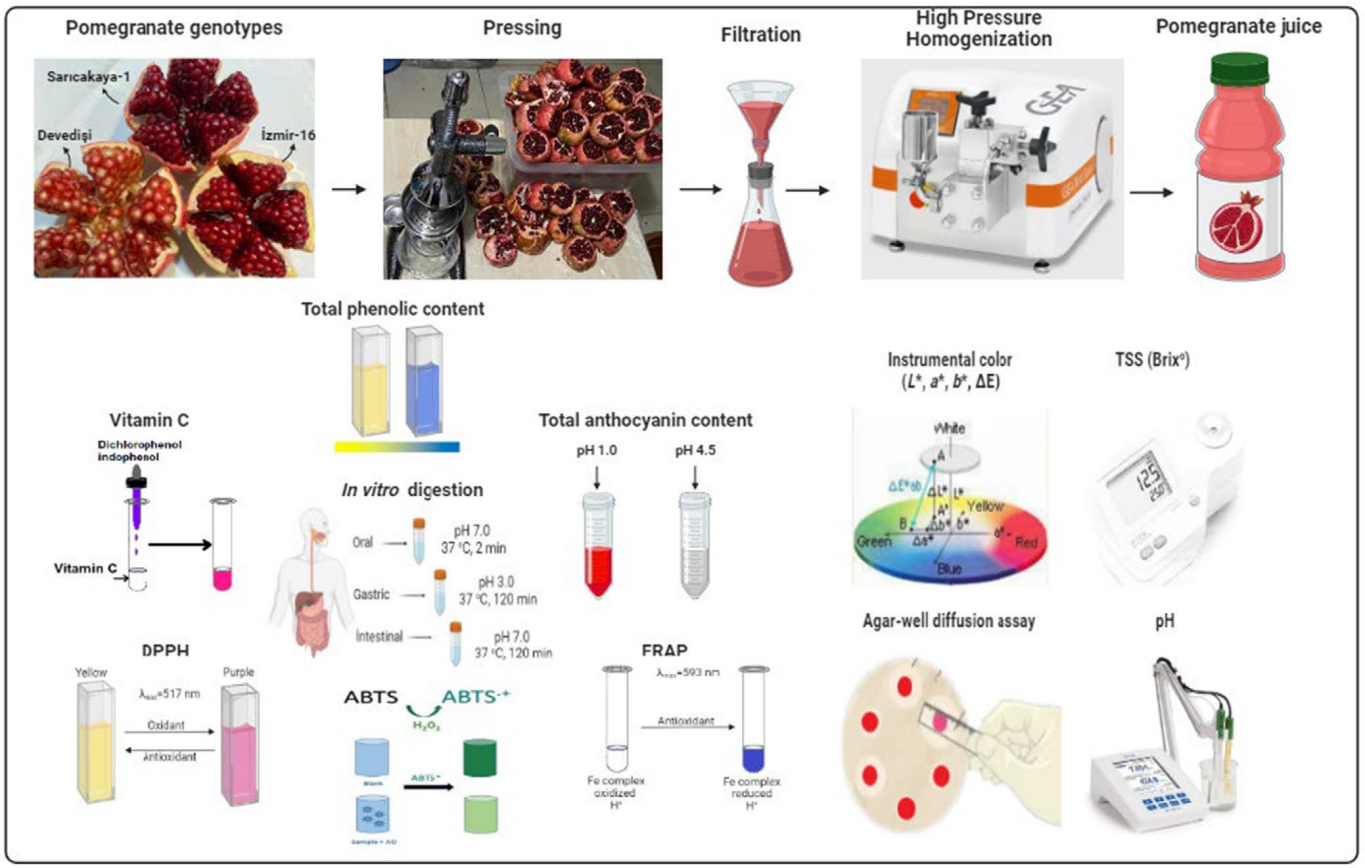
Preparation of HPH‐treated pomegranate juice samples and analyses.

In this study, three pomegranate genotypes (Devedişi, İzmir‐16, and Sarıcakaya‐1) in the sweet group grown in the Central Sakarya basin and in the same commercial garden were used. Some pomological characteristics of PJS obtained from three Turkish genotypes are given in Table S1. Primarily, undamaged fruits were selected and washed with tap water. Freshly squeezed PJS were obtained by pressing technique using a portable juicer and filtered through a filter cloth. After filtration, the PJS were subjected to a single cycle high‐pressure homogenization (50, 100, and 150 MPa) using a table‐top homogenizer (GEA Homogenizer Panda PLUS 2000, Parma, Italy). HPH‐untreated PJS were considered as Control groups. The maximum flow rate in the homogenizer was 9 L/h. The inlet temperature of the juice was about 4°C, while the outlet temperature was in the range of 35.3°C–39.3°C. Therefore, cooling of the PJS in an ice water bath was performed immediately after HPH treatment. All treatments were filled into airtight sterile dark containers and stored at 4°C until the rapid analysis period. The preparation of HPH‐treated PJS and the analyses performed are schematized in Figure 1.

### 2.3 Physicochemical Analysis of PJS

Total soluble solids (TSS) were determined at 20°C with a digital refractometer (Hanna HI 96801, USA). Water activity (*a*_*w*_) values at 20°C were recorded with a temperature‐controlled device (WaterLab Steroglass, Italy). A benchtop pH meter (Hanna Instruments, USA) calibrated with buffer solutions was used for pH measurements. Instrumental color [L* (darkness: L* = 0; lightness: L* = 100), a* (redness: +a*; greenness: −a*), and b* (yellowness: +b*; blueness: −b*)] measurements were performed with a colorimeter (Konica Minolta CR400, Japan) equipped with a D65 illuminant, 11 mm measuring aperture, and 2° standard observer. The overall color change (Δ*E*) was determined by the following equation:

$$\Delta E=\sqrt{{\left(\Delta L^{*}\right)}^{2}+{\left(\Delta \;a^{*}\right)}^{2}+{\left(\Delta \;b^{*}\right)}^{2}}$$

where ΔL*, Δa*, and Δb* indicate the difference between the L*, a*, and b* values of untreated and treated PJ, respectively.

### 2.4 Antimicrobial Activity of PJS

The antibacterial and antifungal activity of PJS was tested by agar‐well diffusion assay using seven bacteria (*Staphylococcus aureus*, *Micrococcus luteus*, *Enterococcus faecalis*, *Escherichia coli*, *Klebsiella pneumoniae*, *Pseudomonas aeruginosa*, *Yersinia enterocolitica*) and one fungus (*Candida albicans*).

The agar‐well diffusion assay was performed as described by Aksu et al. (2023) with minor modifications. First, reactivation of bacterial and fungal strains was performed by transferring from stock cultures to the appropriate medium (BHI broth for bacteria, SD broth for fungi). The bacterial turbidity of the overnight cultures was adjusted to McFarland 0.5 using a densitometer (Biosan Den‐1, Riga, Latvia). An aliquot (100 μL) of each bacterial and fungal inoculum was spread on the surface of MHA and PDA under aseptic conditions. The loading area for the antimicrobial agent was created by punching wells (6 mm in diameter) on the agar in Petri dishes using a sterile cork borer. The wells were directly loaded with PJS (30 μL) and sterilized through a 0.45 μm pore diameter syringe filter. Gentamicin and Amphotericin B were included in the analysis as reference antibiotics for bacteria and fungi, respectively. After inoculation, bacterial and fungal plates were incubated at 37°C for 18 h and 25°C for 48 h, respectively. The zones of inhibition (mm) were recorded using a digital caliper.

### 2.5 In Vitro Gastrointestinal Digestion

The in vitro simulated digestion assay was performed according to the procedures described by Brodkorb et al. (2019) with minor modifications. Simulated salivary fluid (SSF), simulated gastric fluid (SGF), and simulated intestinal fluid (SIF) were prepared according to the specified volumes of electrolyte stock solutions in the procedure. Prior to analysis, pepsin and pancreatin enzyme activities were determined by following the instructions in the protocol. As a first step, the oral phase was carried out by preparing a simulated medium (pH 7.0) containing 5 mL of PJS, 4 mL of SSF, 25 μL of 0.3 M CaCl_2_, and 975 μL of distilled water. The mixture was incubated for 2 min (37°C at 100 rpm) on a rotary shaker. Afterward, 10 mL of the oral bolus was mixed with 8 mL SGF, 0.5 mL of pepsin enzyme solution (2000 U/mL) and 5 μL of 0.3 M CaCl_2_ to simulate gastric digestion. The pH of the mixture was adjusted to 3.0 using 1 M HCl and the total volume was made up to 20 mL with distilled water. The mixture was shaken for 120 min under the same conditions (37°C at 100 rpm). In the final step, intestinal digestion was simulated by mixing 20 mL gastric chyme, 8 mL SIF, 5 mL pancreatin solution (100 U/mL), 0.04 mL 0.3 M CaCl_2_, and 3 mL bile salts (10 mM), 1 M NaOH for pH adjustment and up to 40 mL distilled water. The final mixture was incubated for 2 h at 100 rpm on a rotary shaker at 37°C. To minimize misinterpretations from digestive fluids, a blank digestion was conducted without PJS subjected to the same conditions as above. Following the gastric and intestinal digestion stages, pH adjustments required for enzyme inactivation were performed and the samples were quickly frozen at −20°C. For the analyses, the mixtures were centrifuged at 6000 *g* at 4°C for 20 min and then filtered through a 0.45 μm syringe filter.

### 2.6 Total Phenolic Content (TPC)

The Folin–Ciocalteu method described by Singleton, Orthofer, and Lamuela‐Raventós (1999) was followed in the measurement of the total phenolic content (TPC) of the samples. TPC values were calculated based on the calibration curve plotted using different concentrations of a standard gallic acid solution and reported as milligram gallic acid equivalents (GAE) per liter.

### 2.7 Total Anthocyanin Content (TAC)

Total anthocyanin content (TAC) was assessed by measuring the pH‐dependent change in color of juices exposed to reaction with buffer solutions of 0.025 M potassium chloride (pH 1.0) and 0.4 M sodium acetate (pH 4.5). The results were expressed as milligrams of cyanidin‐3‐glucoside (C3G) per liter (Lee, Durst, and Wrolstad 2005).

### 2.8 Ascorbic Acid Content (AAC)

The AAC of samples was determined by spectrophotometric approach that relied on the decolorization of 2,6‐dichlorophenol indophenol dye in the presence of ascorbic acid. After the dye not reduced by ascorbic acid was extracted with xylene, absorbance was read at 500 nm wavelength. The results were calculated using the calibration curve plotted using different concentrations of standard ascorbic acid solution (Cemeroglu 2010).

### 2.9 Antioxidant Capacity Assays

The antioxidant capacity of the samples was evaluated by three different assays, including radical (DPPH and ABTS) scavenging and ferric‐reducing antioxidant power (FRAP).

DPPH antiradical activity (DPPH‐ARA) analysis of the samples was performed according to Brand‐Williams, Cuvelier, and Berset (1995) with some modifications. Briefly, an aliquot (100 μL) of undigested and digested samples diluted in appropriate proportions was mixed in a test tube with 2.9 mL of DPPH solution (0.1 mM). Thoroughly vortexed mixtures were incubated at 30°C for 30 min in the dark. Absorbance measurements were performed at 517 nm.

ABTS antiradical activity (ABTS‐ARA) analysis was performed following the protocol described by Re et al. (1999). Briefly, ABTS stock solution (7 mM) and potassium persulfate (2.45 mM) were reacted in the dark for 12 h to procure ABTS radical solution (ABTS‐RS). ABTS‐RS was diluted with ethanol until the absorbance was 0.700 ± 0.02 at 734 nm. Subsequently, 50 μL of diluted undigested and digested samples were mixed with ABTS test solution (2950 μL). Absorbance values were measured at 734 nm exactly 6 min after the incubation period.

FRAP analysis was performed following the procedure described by Benzie and Strain (1996) with slight modifications. Just prior to analysis, FRAP reagent was freshly prepared by mixing 10 mM TPTZ (in 40 mM HCl), 20 mM FeCl_3_.6H_2_O, and 0.3 M acetate buffer (pH 3.6) solutions at specified volumetric ratios (1:1:10). The final reaction mixture consisted of an aliquot (0.1 mL) of diluted samples and FRAP reagent (2.9 mL). The absorbance was read at 593 nm in the spectrophotometer after 4 min of reaction at 37°C. The results of three antioxidant capacity assays (DPPH‐ARA, ABTS‐ARA, and FRAP) were presented as mmol Trolox‐equivalent (TE)/L.

### 2.10 Statistical Analysis

All experimental data were analyzed by one‐way ANOVA using SPSS 25.0 software. Tukey's multiple comparison test was used to evaluate significant differences (*p* < 0.05). All analyses were performed in triplicate, and the results were presented as mean ± standard deviation in tables and figures.

## 3 Results and Discussion

### 3.1 pH, Total Soluble Solids (TSS), water Activity (*a*_*w*_), and Instrumental Color Values of PJS

The effects of HPH (50, 100, and 150 MPa) on pH, TSS, and *a*_*w*_ values of PJS obtained from three different genotypes are presented in Table 1. TSS values ranged from 16.17°–18.60°Brix with significant (*p* < 0.05) differences between genotypes and treatments. TSS of PJS for all genotypes was reduced by HPH treatment, most pronounced at 150 MPa pressure. In contrast to the TSS values, HPH treatment increased the pH of PJS with increasing homogenization pressure. Lower pH values were measured in Control samples of all genotypes than in all HPH‐treated samples (*p* < 0.05). On the other hand, Devedişi had lower (*p* < 0.05) pH compared to other genotypes. Regarding the *a*_*w*_ values, the Control sample of İzmir‐16 had lower value than the other genotypes and *a*_*w*_ values generally increased with HPH treatment. Regarding water activity, *a*_*w*_ values were lower (*p* < 0.05) in Control samples of Devedişi and Sarıcakaya‐1 and increased with HPH treatment in these genotypes as opposed to a decrease in İzmir‐16. These differences in basic parameters of juices can be related to genotype, maturity level, processing method, and growing conditions (Topalović et al. 2021). Our findings for pH and TSS were in a similar range to those previously reported for PJS (Ferrara et al. 2011; Tarantino et al. 2022; Mena et al. 2011; Benjamin and Gamrasni 2020).

**TABLE 1:** Physicochemical quality parameters [total soluble solids (TSS), pH, water activity (*a*_*w*_), and instrumental color values] of HPH‐treated pomegranate juices from three different genotypes.

Genotype	Treatment	TSS (°Brix)	pH	*a*_*w*_	L*	a*	b*	∆E*
Devedişi	Control	17.00 ± 0.00^aB^	3.19 ± 0.03^bB^	0.981 ± 0.00^bB^	25.42 ± 0.04^aA^	39.93 ± 0.21^aA^	18.62 ± 0.29^aA^	0 ± 0^dA^
	50 MPa	16.57 ± 0.06^bB^	3.30 ± 0.04^aAB^	0.981 ± 0.00^bA^	25.76 ± 0.11^aA^	39.15 ± 0.05^bA^	18.01 ± 0.15^bA^	1.1 ± 0.08^cC^
	100 MPa	16.43 ± 0.06^cC^	3.33 ± 0.02^aA^	0.992 ± 0.00^aA^	24.82 ± 0.09^bA^	38.39 ± 0.08^cA^	16.76 ± 0.16^cA^	2.49 ± 0.19^bB^
	150 MPa	16.17 ± 0.06^dC^	3.36 ± 0.02^aA^	0.989 ± 0.01^aA^	24.47 ± 0.03^cA^	37.44 ± 0.10^dA^	15.80 ± 0.03^dA^	3.88 ± 0.07^aA^
Izmir‐16	Control	18.60 ± 0.10^aA^	3.26 ± 0.01^bA^	0.985 ± 0.00^aA^	21.15 ± 0.12^aB^	35.95 ± 0.15^aB^	12.21 ± 0.11^aB^	0 ± 0^cA^
	50 MPa	17.87 ± 0.06^bA^	3.33 ± 0.01^aA^	0.980 ± 0.00^bA^	20.46 ± 0.08^bB^	34.12 ± 0.22^bB^	10.68 ± 0.18^bB^	2.49 ± 0.3^bA^
	100 MPa	17.77 ± 0.06^bB^	3.34 ± 0.01^aA^	0.981 ± 0.00^bB^	19.82 ± 0.13^dB^	32.27 ± 0.34^cB^	9.51 ± 0.34^cB^	4.75 ± 0.5^aA^
	150 MPa	17.90 ± 0.00^bA^	3.35 ± 0.01^aA^	0.987 ± 0.00^aA^	20.32 ± 0.04^cB^	33.72 ± 0.14^bB^	11.02 ± 0.18^bB^	2.66 ± 0.21^bB^
Sarıcakaya‐1	Control	18.53 ± 0.06^aA^	3.22 ± 0.02^cAB^	0.980 ± 0.00^bB^	19.89 ± 0.11^aC^	34.12 ± 0.25^aC^	10.52 ± 0.17aC	0 ± 0^cA^
	50 MPa	18.03 ± 0.12^bA^	3.26 ± 0.01^bB^	0.981 ± 0.00^abA^	19.36 ± 0.04^bC^	32.92 ± 0.15^bc^	9.34 ± 0.08^bC^	1.76 ± 0.17^bB^
	100 MPa	18.37 ± 0.12^aA^	3.29 ± 0.01^aB^	0.981 ± 0.00^aA^	18.86 ± 0.02^cC^	31.08 ± 0.12^cC^	8.15 ± 0.09^cC^	3.99 ± 0.14^aA^
	150 MPa	17.47 ± 0.06^cB^	3.30 ± 0.01^aC^	0.982 ± 0.00^aA^	18.88 ± 0.01^cC^	30.73 ± 0.04^cC^	8.25 ± 0.10^cC^	4.20 ± 0.08^aA^

*Note:* Different lowercase letters in the same column indicate significant differences between treatments under the same genotype, while uppercase letters in the same column indicate significant differences between genotypes for the same treatment (*p* < 0.05, Tukey's test).

The L* (lightness), a*(redness), and b* (yellowness) values of PJS obtained from different varieties were in the range of 18.86–25.76, 30.73–39.93, 8.15–18.62, respectively. Instrumental color values (Table 1) were consistent with visually defined colors (Table S1). Devedişi PJS were brighter and redder compared to other genotypes (*p* < 0.05). Depending on the genotype, measuring device, and processing method, the color values of PJS vary in a wide range as reported in the literature (Ozgen et al. 2008; Zaouay et al. 2012; Benjamin and Gamrasni 2020; Yuan et al. 2022). Instrumental color values (L*, a*, and b*) of PJS were generally decreased (*p* < 0.05) by HPH treatment compared to control, with significant differences depending on homogenization pressure. HPH treatment caused a loss of lightness and redness of PJS up to 6.29% and 10.24%, respectively. In general, the lowest (*p* < 0.05) redness and brightness were determined in the samples treated with HPH at 100 MPa and 150 MPa pressure. Compared to untreated juice samples, the overall color change (Δ*E*) in the range of 1.1–4.75 was more visible in 100 MPa (for İzmir‐16) and 150 MPa (for Devedişi and Sarıcakaya‐1) HPH treatments. Anthocyanins are responsible for the attractive red color of PJS. Therefore, the loss of the typical bright redness in PJS after HPH treatment can be attributed to the decrease in TAC (up to 5.66%) of HPH‐treated samples in the present study. Similarly, Kruszewski, Zawada, and Karpiński (2021) found a decrease in anthocyanin content and redness values after HPH for blackcurrant juice. However, previous studies on PJS reported no significant change in color values after HPH (100 and 150 MPa) and HPP (350–550 MPa) treatments (Benjamin and Gamrasni 2020; Yuan et al. 2022). On the other hand, Saricaoglu et al. (2019) reported that HPH treatment (75–125 MPa) increased the L*, a*, and b* values of rosehip nectar as a function of increasing pressure and number of passes and attributed this to the increase in carotenoid content after HPH.

### 3.2 Antimicrobial Activity of PJS

Antimicrobial activity against test microorganisms was evaluated by agar‐well diffusion method and the zones of inhibition around the wells loaded by PJS are presented in Table 2. All treatments were effective against all Gram‐positive bacteria tested, with zones of inhibition ranging from 13.0–17.8 mm. Regarding Gram‐negative bacteria, PJS showed inhibitory activity for *Y. enterocolitica* and *E. coli*, but had no antibacterial effect on *K. pneumoniae* and *P. aeruginosa*. On the other hand, no inhibitory effect of PJS was observed against the growth of *C. albicans* fungus. The highest inhibition zones for Gram‐positive and Gram‐negative bacteria were recorded for *M. Luteus* (17.2–17.8 mm) and *E. coli* (16.3–17.5 mm), respectively. Gentamicin and Amphotericin B were more effective (*p* < 0.05) than PJS against bacteria and fungi for which they were used as reference antibiotics. The antimicrobial activity of PJS was not significantly affected by HPH treatment. Namely, there was no difference (*p* > 0.05) between HPH‐treated and untreated treatments for zones of inhibition on all bacteria tested. Similarly, Alexandre et al. (2019) found that the antimicrobial activity of pomegranate peel against *B. cereus*, *E. coli*, *S. aureus*, *P. aeruginosa*, and several lactic acid bacteria was not significantly changed by exposure to high‐pressure (300 and 600 MPa) extraction. A study evaluating the effect of various processing conditions on PJS exhibited that increasing pressing pressure and yield did not lead to a significant change in the antimicrobial activity of juices (Türkyılmaz et al. 2013).

**TABLE 2:** Inhibition zones (mm) on the tested microorganisms expressing the antimicrobial activity of HPH‐treated pomegranate juices of three different genotypes.

	Treatment	Gram‐negative bacteria				Gram‐positive bacteria			Fungus
		YE	KP	EC	PA	ML	EF	SA	CA
Devedişi	Control	10.2 ± 0.1^b^	—	16.6 ± 0.6^b^	—	17.8 ± 0.5^b^	13.3 ± 0.8^b^	14.3 ± 1.2^b^	—
	50 MPa	10.0 ± 0.3^b^	—	16.3 ± 0.4^b^	—	17.5 ± 0.7^b^	13.0 ± 1.0^b^	14.2 ± 1.3^b^	—
	100 MPa	10.2 ± 0.4^b^	—	16.8 ± 0.5^b^	—	17.5 ± 0.6^b^	13.2 ± 1.1^b^	14.2 ± 1.1^b^	—
	150 MPa	10.3 ± 0.2^b^	—	16.5 ± 0.7^b^	—	17.7 ± 0.6^b^	13.0 ± 1.1^b^	14.2 ± 1.2^b^	—
Izmir‐16	Control	10.7 ± 0.0^b^	—	16.9 ± 0.5^b^	—	17.2 ± 0.5^b^	13.6 ± 0.4^b^	14.3 ± 1.0^b^	—
	50 MPa	10.9 ± 0.2^b^	—	16.6 ± 0.7^b^	—	17.2 ± 0.6^b^	13.7 ± 0.6^b^	14.3 ± 1.0^b^	—
	100 MPa	10.9 ± 0.2^b^	—	16.8 ± 0.8^b^	—	17.3 ± 0.2^b^	13.6 ± 0.6^b^	14.3 ± 1.1^b^	—
	150 MPa	10.9 ± 0.4^b^	—	16.6 ± 0.6^b^	—	17.2 ± 0.5^b^	13.6 ± 0.6^b^	14.3 ± 1.1^b^	—
Sarıcakaya‐1	Control	11.2 ± 0.6^b^	—	17.5 ± 0.6^b^	—	17.5 ± 0.6^b^	13.6 ± 0.6^b^	15.7 ± 1.0^b^	—
	50 MPa	11.2 ± 0.3^b^	—	17.4 ± 0.4^b^	—	17.5 ± 0.9^b^	13.6 ± 0.6^b^	15.7 ± 1.1^b^	—
	100 MPa	11.1 ± 0.0^b^	—	17.3 ± 0.4^b^	—	17.6 ± 0.9^b^	13.6 ± 0.8^b^	15.8 ± 0.9^b^	—
	150 MPa	11.1 ± 0.3^b^	—	17.3 ± 0.5^b^	—	17.6 ± 0.7^b^	13.7 ± 0.6^b^	15.8 ± 0.7^b^	—
	Gentamicin (10 μg/disc)	19.3 ± 0.9^a^	22.4 ± 0.8^a^	26.0 ± 0.6^a^	22.1 ± 0.4^a^	25.6 ± 0.6^a^	20.7 ± 0.4^a^	27.8 ± 0.5^a^	NT
	Amphotericin B (100 μg/disc)	NT	NT	NT	NT	NT	NT	NT	19.9 ± 1.06

*Note:* Different lowercase letters in the same column indicate significant differences between all treatments (*p* < 0.05, Tukey's test).

Abbreviations: “–”, no activity; CA, *Candida albicans* (ATCC10231); EC, *Escherichia coli* (ATCC25922); EF, *Enterococcus faecalis* (ATCC29121); KP, *Klebsiella pneumoniae* (ATCC13883); ML, *Micrococcus luteus* (NRRL B‐1018); NT, not tested; PA, *Pseudomonas aeruginosa* (ATCC27853); SA, *Staphylococcus aureus* (ATCC6538); YE, *Yersinia enterocolitica* (ATCC27729).

The degree of antibacterial activity depends on several parameters, including variety, fruit parts, pH, geographical origin, bioactive component content, as well as extraction and processing methods (Pagliarulo et al. 2016; Türkyılmaz et al. 2013; Opara, Al‐Ani, and Al‐Shuaibi 2009). Similar to our results, previous studies have reported antimicrobial activity of PJS and extracts of different parts (aril, peel, pericarp, etc.) of pomegranate (Celiksoy and Heard 2021; Pagliarulo et al. 2016; Türkyılmaz et al. 2013; Opara, Al‐Ani, and Al‐Shuaibi 2009; Duman et al. 2009). Pagliarulo et al. (2016) reported that both juice and peel extracts of pomegranate had antibacterial effects on *S. aureus* and *E. coli* at levels competitive with the reference antibiotic (ampicillin). The antimicrobial activity of pomegranate has been associated with bioactive compounds such as phenolic acids, anthocyanins, tannins, punicalagin as well as acidic pH. Tannins exhibit antimicrobial activity by altering cell permeability and substance transfer, inhibiting important bacterial enzymes, and forming stable complexes with metal ions (Türkyılmaz et al. 2013). The inhibitory effect of phenolic compounds on microorganisms is based on many mechanisms such as membrane destruction, reduction of intracellular pH, complexation with metal ions, enzyme malfunction, and damage to the microbe's respiratory chain. Furthermore, phenolics react with sulfhydryl groups of proteins, making them unavailable for enzymes and microbial growth (Celiksoy and Heard 2021). Opara, Al‐Ani, and Al‐Shuaibi (2009) found a strong correlation between the antibacterial activity of pomegranate aril and peel extracts and the pH of these fractions. Duman et al. (2009) reported that dark‐colored pomegranate cultivars with high acidity and rich anthocyanin content had higher antimicrobial potency. Accordingly, the inhibitory effect shown in the present study on certain test microorganisms can be attributed to the acidic pH (ranging from 3.19–3.36) and phenolic constituents of PJS, including anthocyanins.

### 3.3 Total Phenolic Content

**FIGURE 2:**
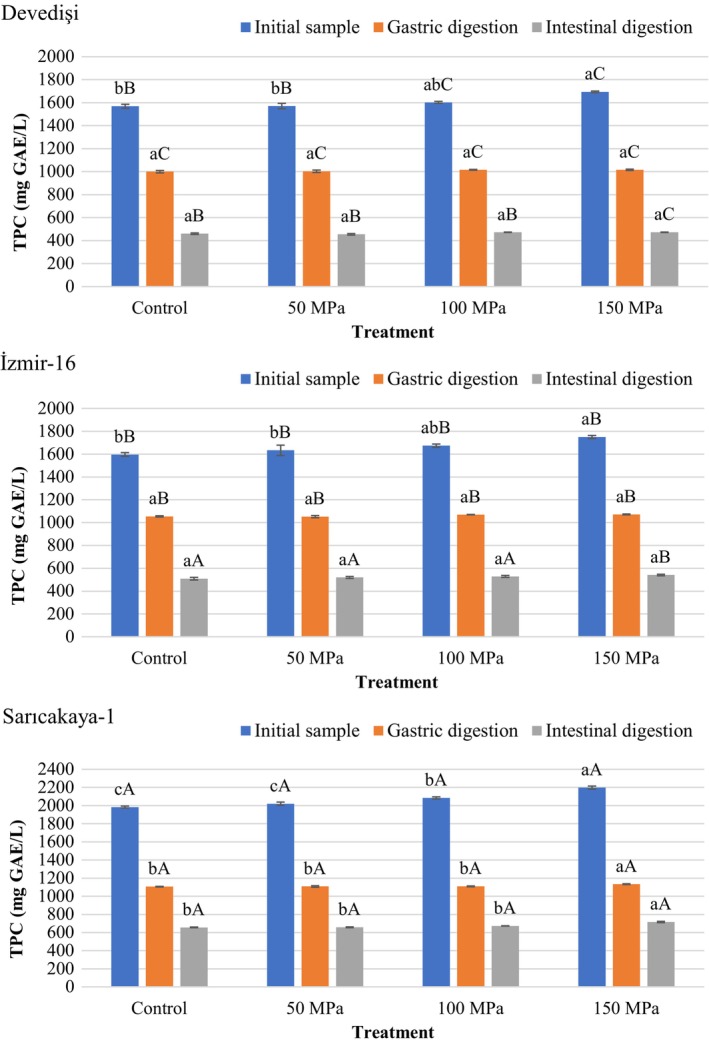
Changes in total phenolic content (TPC) of HPH‐treated pomegranate juices obtained from different genotypes during in vitro simulated digestion. Bar graphs with different letters indicate significant differences between treatments (a–c) within each genotype or between genotypes (A–C) for the same treatment (*p* < 0.05, Tukey's test).

As shown in Figure 2, the TPC of Control and HPH‐treated PJS obtained from three Turkish genotypes ranged from 1569.22 to 2198.91 mg GAE/L at the initial stage. The highest initial TPC values were determined in Sarıcakaya‐1 (1984.06–2198.91 mg GAE/L), while Devedişi (1569.22–1694.53 mg GAE/L) had the lowest values (*p* < 0.05). Our results were in agreement with previous studies (Mena et al. 2011; Zaouay et al. 2012; Ozgen et al. 2008) reporting similar TPC for PJS, but lower than findings reported by some researchers (Yıkmış et al. 2022; Benjamin and Gamrasni 2020; Desseva and Mihaylova 2019). These differences can be attributed to many factors such as cultivar, growing area, agricultural practices, climate and soil characteristics, fruit maturity stages, as well as processing and storage (Gundogdu and Yilmaz 2012; Kalaycioglu and Erim 2017). Anthocyanins, ellagitannins, flavanols, flavones, hydroxybenzoic acids, hydroxycinnamic acids, and derivatives of these compounds are phenolics identified for pomegranate. Especially anthocyanins and ellagitannins (such as ellagic acid and punicalagin) contribute to many bioactive properties of pomegranate including antioxidant and antimicrobial properties (Topalović et al. 2021; Mena et al. 2011; Vegara et al. 2014).

HPH treatment increased the initial TPC of PJS. Regardless of genotype, a significant increase of 7.97%–10.83% was observed in the initial TPC of 150 MPa HPH‐treated PJS compared to Control (unprocessed) samples (*p* < 0.05), while the TPC of samples treated at 50 MPa pressure was similar to Control (*p* > 0.05). In contrast to the other genotypes, the increase (5.12%) in initial TPC of PJS treated with 100 MPa HPH compared with the Control was significant (*p* < 0.05) only in Sarıcakaya‐1. The initial TPC of Devedişi and İzmir‐16 were similar (*p* > 0.05) for Control and 50 MPa HPH treatments. However, Sarıcakaya‐1 had the highest (*p* < 0.05) initial TPC values for all treatments. These results are similar to Benjamin and Gamrasni (2020) who reported an increase in TPC of PJS treated with HPH. A previous study evaluating the effect of HPH on strawberry juice determined that there was no difference in TPC between the Control and samples homogenized at 60 MPa pressure, but HPH of 100 MPa significantly increased TPC (Karacam, Sahin, and Oztop 2015). Varela‐Santos et al. (2012) reported that the TPC of PJS increased up to 16.87% with HPP applied for different times in the pressure range of 350–550 MPa. HPH treatment is considered to favor the release of bound phenolic substances by weakening cell walls and disrupting cell structure, thereby increasing phenolic content (He et al. 2016). On the other hand, the extractability and stability of phenolic compounds in fruit juices varies depending on the pressure applied during HPH processing and the number of passes (Yong, Song, and Choo 2021).

In vitro gastrointestinal digestion negatively affected the TPC of PJS, regardless of genotype and HPH treatment. After gastric digestion, the TPC of PJS obtained from Devedişi, İzmir‐16, and Sarıcakaya‐1 decreased by 36.12%–40.05%, 33.96%–38.81% and 44.25%–48.46%, respectively. A similar trend of decrease in TPC of PJS after in vitro gastric digestion was determined by Yıkmış et al. (2022). On the other hand, a remarkable decrease of 66.90%–72.12% was observed in the TPC of PJS from three genotypes after intestinal digestion. As a result, a maximum of 33.10% of TPC remained after digestion. These results indicated that phenolics was unstable under gastric and intestinal conditions. On the other hand, the recoveries of TPC were similar in HPH‐treated and untreated samples after gastric and intestinal digestion (*p* > 0.05). The descending sort for bioaccessibility of phenolics Sarıcakaya‐1 (32.26%–33.10%) > İzmir‐16 (30.90%–31.89%) > Devedişi (27.88%–29.45%). Marszałek et al. (2023) found that the bioavailability of polyphenols in HPH‐treated apple juice was 17% higher than in unprocessed juice, which can be attributed to better extraction of polyphenols and greater absorption into micelles as a result of the reduction in particle size of the juices due to HPH treatment. Our results were consistent with those of Desseva and Mihaylova (2019), who determined a dramatic 75% loss of TPC from PJS after in vitro gastrointestinal digestion. Similarly, Mihaylova et al. (2021) determined the bioaccessibility of TPC in PJS subjected to in vitro simulated gastrointestinal digestion as 19.74%. Pérez‐Vicente, Gil‐Izquierdo, and García‐Viguera (2002) determined the available TPC of PJS as 29% in the dialyzed fraction after intestinal digestion. He et al. (2016) found that the TPC of grape and orange juices was significantly increased by HPH treatment, but there was no difference between TPC recovered from HPH‐treated and untreated juice samples after in vitro intestinal digestion.

Besides the activity of digestive enzymes and a large pH change in the simulated gastrointestinal tract can be expressed as responsible for the loss in TPC (Marszałek et al. 2023). Polymerization, epimerization, and auto‐oxidation are reactions that cause degradation of phenolics during intestinal digestion (Quan et al. 2020). Polyphenols are very sensitive to pHs close to alkaline, such as in the intestinal tract, and these conditions result in the conversion of some of the compounds into structural forms with different chemical properties. In addition, the interaction of phenolic compounds with other released constituents such as proteins, minerals, or dietary fiber during digestion can lead to loss of phytochemicals (Mihaylova et al. 2021; He et al. 2016).

### 3.4 Total Anthocyanin Content

**FIGURE 3:**
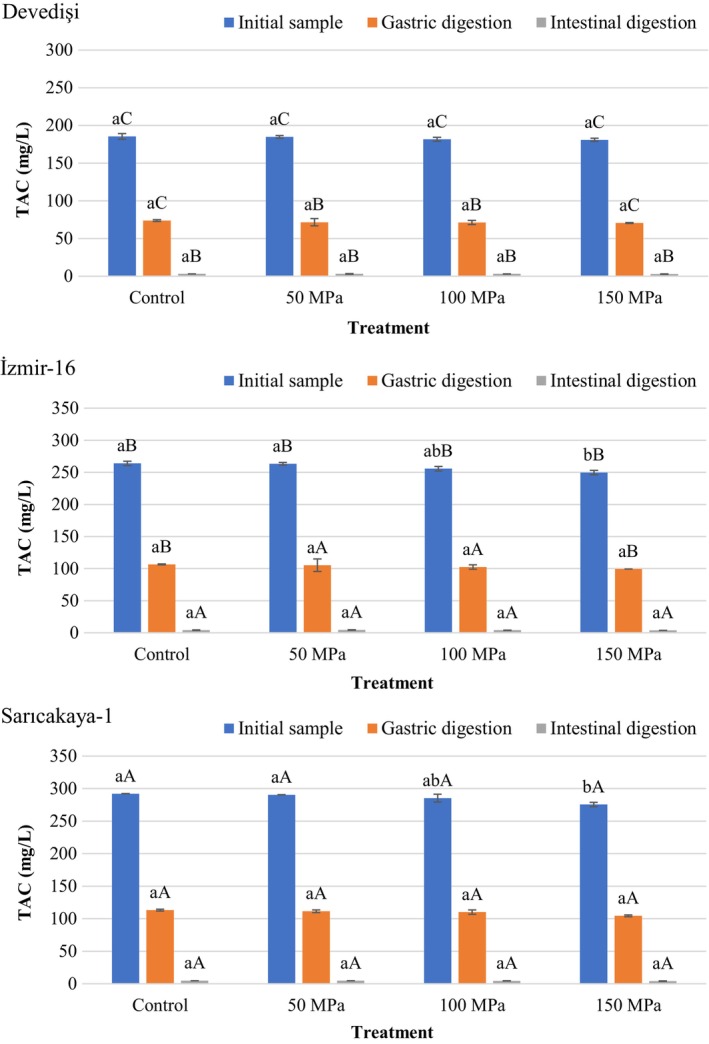
Changes in total anthocyanin content (TAC) of HPH‐treated pomegranate juices obtained from different genotypes during in vitro simulated digestion. Bar graphs with different letters indicate significant differences between treatments (a–b) within each genotype or between genotypes (A–C) for the same treatment (*p* < 0.05, Tukey's test).

The changes in TAC of PJS with or without HPH treatment before and after in vitro digestion are displayed in Figure 3. The initial TAC values of the Control PJS from Devedişi, İzmir‐16 and Sarıcakaya‐1 were 185.36, 264.01, and 292.07 mg C3G/L, respectively, which are consistent with the TAC reported for PJS in previous studies (Ozgen et al. 2008; Mena et al. 2011; Zaouay et al. 2012; Topalović et al. 2021). Treatments prepared from Sarıcakaya‐1 (275.53–292.07 mg C3G/L) had a significantly higher (*p* < 0.05) initial TAC compared to those prepared from Devedişi (180.85–185.36 mg C3G/L) and İzmir‐16 (249.65–264.01 mg C3G/L). These results clearly showed that the TAC of PJS depends on the genotype. Anthocyanins are responsible for the color of PJS, and the most predominant anthocyanins in PJS are the glycosides of delphinidin (delphinidin‐3‐glucoside; delphinidin 3,5‐diglucoside) and cyanidin (cyanidin‐3‐glucoside; cyanidin 3,5‐diglucoside), with pelargonidin present in lower concentration (Topalović et al. 2021; Mena et al. 2011). The anthocyanin profile and stability of PJS varies depending on the cultivar, maturity level, processing method (freshly squeezed, extraction, clarification, etc.), agricultural and environmental conditions as well as internal factors (pH, temperature, light, oxygen, enzymes, ascorbic acids, sugars, metals ions, and copigmentation) (Topalović et al. 2021; Mena et al. 2011).

Regardless of genotype, HPH treatment reduced the initial TAC up to 5.66% compared to the control in a pressure‐dependent manner, but this reduction was significant (*p* < 0.05) only for 150 MPa HPH‐treated İzmir‐16 and Sarıcakaya‐1 samples. HPH applied at low pressure (50 MPa) did not cause a significant change (*p* > 0.05) in the initial TAC of PJS of the three genotypes. Similar to the present study, previous studies have shown that TAC is generally stable against HPH treatment, except at high pressure. In this regard, a study on bilberry extract (pH 3.5) revealed that mechanical stresses had an insignificant effect on anthocyanin stability even at HPH treatments up to 150 MPa, and the anthocyanin content remained almost constant regardless of the initial concentration of the extract (Frank, Köhler, and Schuchmann 2012). On the other hand, increasing the temperature during homogenization is a negative aspect of HPH as it can affect the stability of anthocyanins (Marszałek et al. 2017). In the present study, the reduction in anthocyanin content up to a maximum of 5.66% by HPH treatment can be attributed to the product outlet temperature rising up to 39.3°C. Also, the presence of ascorbic acid (7.96–16.21 mg/100 mL) in PJS may be responsible for this limited decrease in anthocyanin content even at 150 MPa pressure. Yu et al. (2014) reported a 38.8% decrease in C3G content after treatment with HPH (one pass at 200 MPa) of fresh mulberry juice without added ascorbic acid and related this reduction to the effect of peroxidase or polyphenol oxidase in the absence of ascorbic acid in the medium.

As reflected in Figure 3, the TAC in PJS was significantly reduced by gastric and intestinal digestion. After gastric digestion, the reduction in the TAC of PJS compared to the initial content was in the range of 59.62%–62.11%. These results were consistent with Yıkmış et al. (2022) and Yuan et al. (2022), who reported a similar decrease in TAC of PJS after gastric digestion. In contrast, Pérez‐Vicente, Gil‐Izquierdo, and García‐Viguera (2002) observed a slight increase (10%) in the initial anthocyanin concentration (141 mg/L) of PJS (pH 3.8) after stomach digestion and attributed this increase to the pH of the simulated stomach fluid (pH 2) causing an increase in the flavylium cation. This difference may be due to the close pH of the original PJS (3.19–3.36) and the gastric digestive fluid (pH 3.0) in the present study. As shown in Figure 3, all treatments had significantly (*p* < 0.05) lower TAC after gastrointestinal digestion compared to initial samples. TAC values recovered after intestinal digestion for Devedişi, İzmir‐16, and Sarıcakaya‐1 were 2.97–3.21 mg C3G/L, 4.01–4.57 mg C3G/L, and 4.21–4.81 mg C3G/L, respectively. In other words, TAC bioavailability of PJS after simulated digestion was 1.53%–1.74%. There was no significant difference (*p* > 0.05) between the HPH‐treated and untreated samples of all genotypes in terms of TAC recovered after gastric and intestinal digestions. In agreement with our results, Yuan et al. (2022) found that 63% and 99% loss in TAC of PJS occurred after gastric and intestinal digestion, respectively, and only 2 mg/L (bioaccessibility 1%) anthocyanin was recovered after gastrointestinal digestion. Pérez‐Vicente, Gil‐Izquierdo, and García‐Viguera (2002) reported that the recovery of TAC in dialyzed and non‐dialyzed fractions after small intestinal digestion of PJS was 2.4% and 15.3%, respectively. Yıkmış et al. (2022) determined the loss of TAC in PJ as 89% after gastrointestinal digestion. Anthocyanins are pigments that are sensitive to pH‐dependent structural changes and are more stable at acidic pH but degrade under neutral conditions. Bio‐transformation of anthocyanins begins with the digestion of food in the oral cavity (optimum pH 5.6–7.9). The gastric fluid (pH 1.5–3.5) offers optimal conditions for the maintenance of flavylium cation and quinoidal blue species. The intestinal phase (pH 6.7–7.4) results in the completion of the bio‐transformation of anthocyanins to low molecular weight molecules (such as phenolic acids and catechol) (Braga et al. 2018).

### 3.5 Ascorbic Acid Content

**FIGURE 4:**
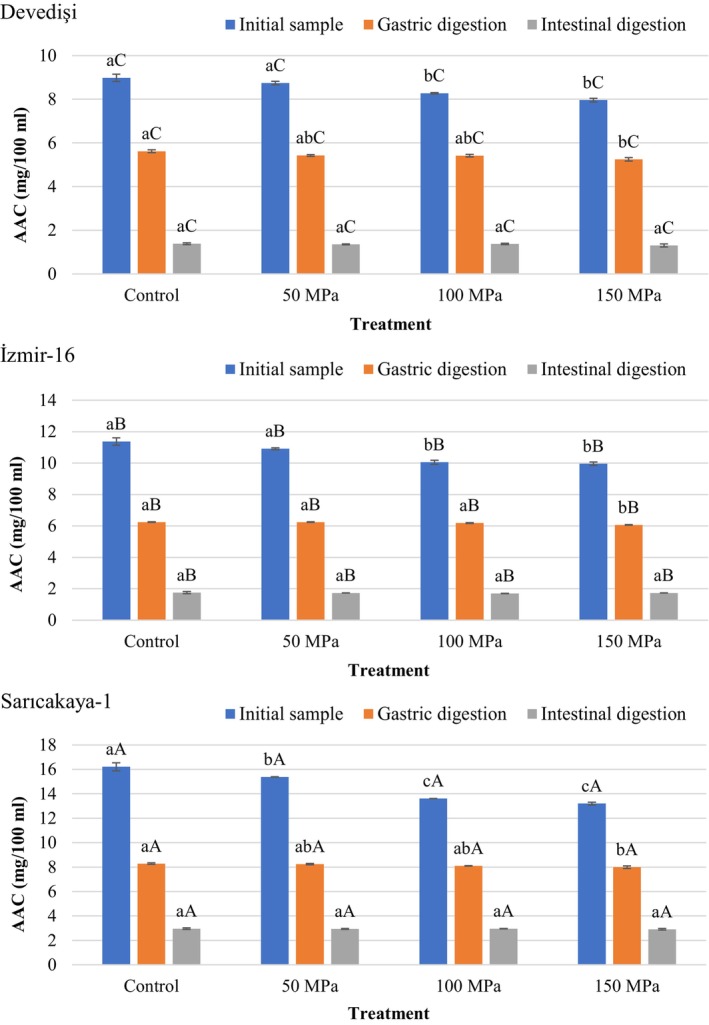
Changes in ascorbic acid content (AAC) of HPH‐treated pomegranate juices obtained from different genotypes during in vitro simulated digestion. Bar graphs with different letters indicate significant differences between treatments (a–c) within each genotype or between genotypes (A–C) for the same treatment (*p* < 0.05, Tukey's test).

Figure 4 presents the AAC of HPH‐treated and untreated PJS before and after in vitro digestion. The AAC in fresh and HPH‐treated PJS ranged from 7.96 to 16.21 mg/100 mL, with significant differences between the genotypes. The highest and lowest values were determined in Sarıcakaya‐1 (13.20–16.21 mg/100 mL) and Devedişi (7.96–8.98 mg/100 mL) PJS, respectively (*p* < 0.05). These values are within the range of values previously reported for PJS by various researchers (Tarantino et al. 2022; Topalović et al. 2021; Ferrara et al. 2011; Mena et al. 2011).

Vit C is one of the most sensitive vitamins and is easily destroyed by oxidation during processing and storage. In this regard, the Vit C content was significantly influenced by the HPH applied to PJS. For all three genotypes, HPH significantly reduced the Vitamin C content (up to 18.57%) of PJS compared to the Control samples. Regardless of genotype, increasing homogenization pressure resulted in greater loss of Vit C content, with the highest reduction (from 11.36% to 18.87%) at 150 MPa HPH treatments (*p* < 0.05). In other words, the highest Vit C content was determined in the Control samples of all genotypes. The loss of vitamin C content in juices from various fruits after HPH treatment is evident from previous studies. Velázquez‐Estrada et al. (2013) described a reduction of 2%, 5%, and 11% in the AAC of orange juice processed with HPH at 100, 200, and 300 MPa, respectively. Benjamin and Gamrasni (2020) found that the AAC of PJS treated with 150 MPa HPH decreased slightly compared to the Control. On the other hand, many studies have shown that HPH retains the AAC of fruit juices better than thermally processed samples (Suárez‐Jacobo et al. 2011; Kruszewski, Zawada, and Karpiński 2021; Yildiz 2019; Velázquez‐Estrada et al. 2013). Saricaoglu et al. (2019) reported that AAC in rosehip nectars treated with HPH at 75, 100, and 125 MPa pressures decreased between 16.91%–36.55% depending on the number of passes, and attributed this reduction to the increase in the final temperature (up to 49.55°C) of the product after HPH treatment. Accordingly, in the present study, the outlet temperature (maximum 39.3°C) of PJS after HPH treatment may also be responsible for the loss of ascorbic acid. In addition, some sample processing conditions such as lack of deaeration of the juice before processing, the presence of oxygen, high shear and temperature, as well as the presence of beryllium‐copper seals of HPH equipment subject to erosion can cause ascorbic acid oxidation (Tribst et al. 2011).

The results presented in Figure 4 showed that the Vit C content gradually decreased after each phase of simulated gastrointestinal digestion. After gastric digestion, a significant loss (ranging from 34.05% to 48.92%) was observed compared to the initial AAC content of the samples (*p* < 0.05). On the other hand, the recovery of ascorbic acid in Devedişi, İzmir‐16, and Sarıcakaya‐1 PJS after in vitro gastrointestinal digestion ranged between 1.30–1.38 mg/100 mL, 1.70–1.75 mg/100 mL and 2.91–2.96 mg/100 mL, respectively. In other words, the loss of AAC after the intestinal phase was between 77.96%–84.68%. Considering the initial values, ascorbic acid bioaccessibility was in the range 15.32%–22.04% depending on genotype and treatment, and the samples presenting the highest bioaccessibility values were Sarıcakaya‐1 (18.23%–22.04%) > İzmir‐16 (15.39%–17.33%) > Devedişi (15.32%–16.57%). Furthermore, the 100 MPa and 150 MPa HPH treatments had the highest (*p* < 0.05) AAC bioaccessibility except for the Devedişi genotype, where all treatments presented similar (*p* > 0.05) recoveries. For all genotypes, the highest and lowest recovery of ascorbic acid in PJS after gastric digestion was in Control (5.62–8.28 mg/100 mL) and 150 MPa HPH‐treated (5.25–7.99 mg/100 mL) samples, respectively (*p* < 0.05). However, looking at the results for three genotypes, there was no significant difference (*p* > 0.05) between treatments in terms of AAC retained after the intestinal phase. These results showed that ascorbic acid is not stable under gastric and intestinal conditions. Ascorbic acid is a heat‐sensitive compound that is easily degraded by chemical and enzymatic oxidation. Oxygen availability, pH changes, temperature, light, enzyme activity, formation of complexes with other components, and the presence of metal ions and reducing agents are factors that promote the degradation of ascorbic acid (Rodríguez‐Roque et al. 2013; Cilla et al. 2011). Ascorbic acid is more stable in acidic media than in alkaline conditions. Furthermore, acidic and low‐temperature environments are more effective than alkaline and high‐temperature conditions to maintain the AAC (Farah et al. 2020). In this regard, changes in pH (adjusted to 7.0, 3.0, and 7.0 for the oral, gastric, and intestinal phases, respectively), the presence of oxygen, and the temperature of the digestive medium (37°C) may be responsible for the significant loss of AAC after in vitro simulated digestion. Our AAC results are consistent with previous studies on juices subjected to in vitro gastrointestinal digestion, which showed significant reductions in AAC. Pérez‐Vicente, Gil‐Izquierdo, and García‐Viguera (2002) found that the loss of ascorbic acid after gastric and intestinal digestion of PJS was 29% and > 80%, respectively. In a previous study on freshly squeezed PJS, Yıkmış et al. (2022) reported that recovery of ascorbic acid in gastric digesta was low and even ascorbic acid was not detected in intestinal digesta. Similar results were reported by Rodríguez‐Roque et al. (2013), who found that ascorbic acid was not stable in the intestinal phase and the bioavailability of a blended juice containing orange, pineapple, and kiwi was 15%. Cilla et al. (2011) determined the loss of ascorbic acid as 16.3%–56% after in vitro digestion in grape, orange, and apricot‐based fruit beverages with or without iron, zinc, and milk.

### 3.6 Antioxidant Capacity

**FIGURE 5:**
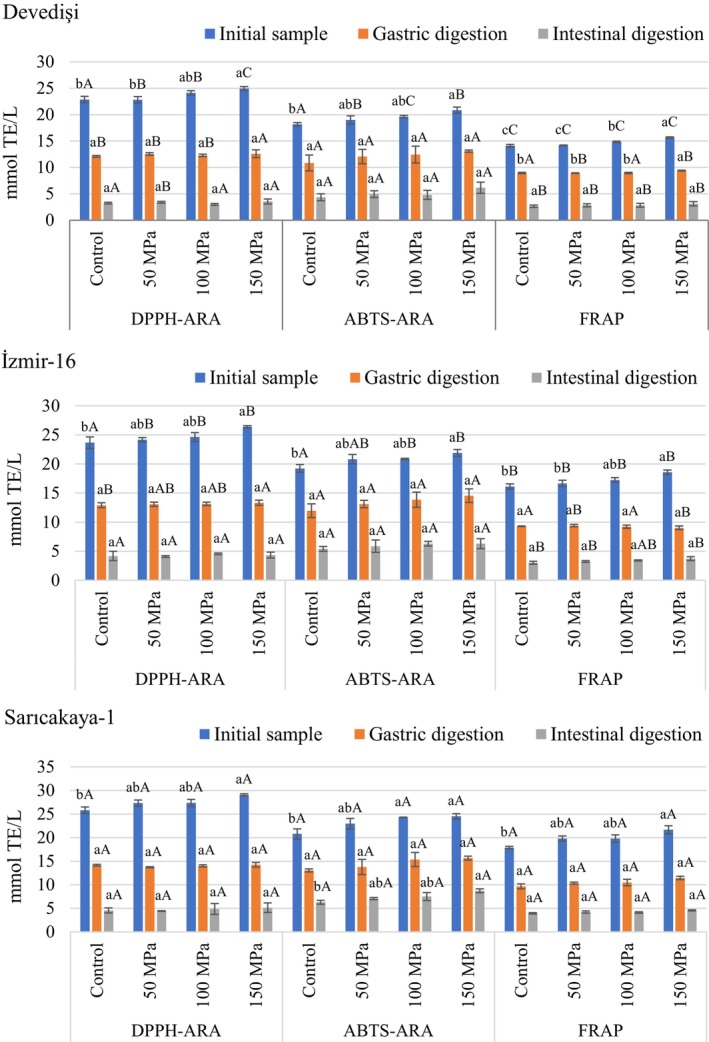
Changes in antioxidant capacity of HPH‐treated pomegranate juices obtained from different genotypes during in vitro simulated digestion. Bar graphs with different letters indicate significant differences between treatments (a–c) within each genotype or between genotypes (A–C) for the same treatment (*p* < 0.05, Tukey's test).

The antioxidant capacity results of HPH‐treated and untreated PJS evaluated using three different assays (DPPH‐ARA, ABTS‐ARA, and FRAP) before and after simulated digestion are given in Figure 5. The initial DPPH‐ARA and ABTS‐ARA values of the HPH‐untreated (Control) juice samples of the three pomegranate genotypes ranged from 22.88–25.83 and 18.18–20.80 mmol TE/L, respectively, with no significant differences (*p* > 0.05) among genotypes. However, a significant variation (*p* < 0.05) was observed in the initial FRAP values of the Control samples depending on the genotypes, with values ranging from 14.13 mmol TE/L (Devedişi) to 17.88 mmol TE/L (Sarıcakaya‐1). These results were within the range reported for PJS (Mena et al. 2011; Zaouay et al. 2012; Vegara et al. 2014), but also differed from the lower or higher values found by some studies in the literature (Yuan et al. 2022; Desseva and Mihaylova 2019; Gundogdu and Yilmaz 2012; Ozgen et al. 2008). Pre‐ and post‐harvest factors such as cultivar, location, climate, maturity level, cultural practice, processing technology, and storage conditions can be considered responsible for these differences (Çam, Hışıl, and Durmaz 2009; Kalaycioglu and Erim 2017).

Considering the results for three genotypes, antioxidant capacity was gradually increased by HPH treatment, depending on the level of pressure applied during HPH (*p* < 0.05). A significant increase (*p* < 0.05) in DPPH‐ARA, ABTS‐ARA, and FRAP values of all samples treated with 150 MPa HPH was observed compared to the Control samples, ranging from 9.13%–12.54%, 13.89%–17.88% and 10.83%–21.20%, respectively. Regarding FRAP values, the hierarchy of genotypes for antioxidant capacity of all treatments was as follows: Sarıcakaya‐1 > İzmir‐16 > Devedişi (*p* < 0.05). Sarıcakaya‐1 had the highest (*p* < 0.05) initial DPPH‐ARA and ABTS‐ARA values for all HPH‐treated samples, while these values were generally similar for Devedişi and İzmir‐16. On the other hand, there was no significant difference (*p* > 0.05) between Control and 50 MPa HPH‐treated samples of all genotypes for antioxidant capacity based on three different assays. In other words, homogenization applied at low pressure (50 MPa) did not significantly change the antioxidant capacity of PJS. A similar trend was also reported by Karacam, Sahin, and Oztop (2015), who described that treatment with 100 MPa HPH significantly increased (around 22%) the DPPH activity of strawberry juice, in contrast to the insignificant effect of HPH applied at 60 MPa pressure. Also, the present results were consistent with previous studies reporting an increase in antioxidant capacity after HPH treatment in similar products such as peach (Yildiz 2019), rosehip nectar (Saricaoglu et al. 2019), pomelo and kiwi juices (Quan et al. 2020). Similarly, Benjamin and Gamrasni (2020) reported that the DPPH activity of PJS was significantly increased by 100 and 150 MPa HPH treatments compared to the untreated Control. According to the present results, HPH treatment increased the antioxidant capacity of PJS, although it decreased the content of antiradical bioactive compounds such as anthocyanins and ascorbic acid. One possible explanation for this increase is that HPH can disrupt the cell walls, allowing more antioxidant compounds to be released (Quan et al. 2020). Hydrolyzable tannins, anthocyanins, ellagic acid derivatives as well as punicalagin derived from the peels are the main phytochemicals that contribute to the total antioxidant capacity of PJS (Tezcan et al. 2009; Varela‐Santos et al. 2012). On the other hand, it is known that there is a strong positive correlation between TPC and antioxidant activity (Çam, Hışıl, and Durmaz 2009). Therefore, the increase in TPC content and extractability of other antioxidant compounds by HPH may have contributed significantly to the enrichment of antioxidant capacity in the present study.

Changes in antioxidant capacity as a result of the in vitro gastrointestinal digestion are displayed in Figure 5. In vitro simulated digestion had a significant negative effect on the antioxidant capacity of all tested treatments. The loss of DPPH‐ARA, ABTS‐ARA, and FRAP values of PJS after gastric phase varied between 44.90%–50.95%, 33.62%–40.26%, and 36.82%–51.37%, respectively, depending on the genotype. In the same vein, DPPH‐ARA, ABTS‐ARA, and FRAP values recovered after intestinal digestion were 12.47%–18.40%, 23.97%–35.61%, and 18.72%–22.11%, respectively, and this variation was genotype‐dependent. Namely, there was a remarkable loss of 64.39%–87.53% in antioxidant capacity after intestinal digestion. This can be attributed to the negative reflection of loss of bioactive compounds such as phenolics and ascorbic acid on the remaining antioxidant capacity after in vitro simulated digestion. Many of the bioactive compounds with antioxidant activity are unstable under intestinal conditions and these compounds can be transformed into other substances with different chemical and physical properties under the action of alkaline pH as well as digestive enzymes (Rodríguez‐Roque et al. 2013; Quan et al. 2020).

For all three genotypes, all treatments had similar (*p* > 0.05) DPPH‐ARA, ABTS‐ARA, and FRAP values after gastric digestion, except for Devedişi, where the 150 MPa HPH treatment had the highest (*p* < 0.05) FRAP values compared with the other treatments. After gastric digestion, all genotypes had similar (*p* > 0.05) ABTS‐ARA values for the same treatment, whereas Sarıcakaya‐1 generally had the highest values for DPPH‐ARA and FRAP. On the other hand, there was no significant (*p* > 0.05) difference between treatments for all three antioxidant capacity assays in the intestinal digesta of all genotypes except ABTS‐ARA values of Sarıcakaya‐1, in which the highest (*p* < 0.05) activity was found in 150 MPa HPH treatment compared with the Control. In addition, ABTS‐ARA and DPPH‐ARA values maintained after intestinal digestion for the same treatment were similar between genotypes, but the highest values for FRAP were in Sarıcakaya‐1.

Our results are consistent with previous studies in which significant reductions in the antioxidant capacity of similar products subjected to in vitro digestion were observed (Mihaylova et al. 2021; Quan et al. 2020; Rodríguez‐Roque et al. 2013). Desseva and Mihaylova (2019) reported that the antioxidant activity of PJS evaluated by four different methods including DPPH, ABTS, CUPRAC, and FRAP decreased significantly (about 70%–80%) after in vitro digestion. In another study conducted on PJS, the recovered DPPH and CUPRAC values after in vitro digestion were between 26%–35% and 25.36%–32.59%, respectively (Yıkmış et al. 2022). In a study comparing the effects of HPP and thermal treatments on PJS, Yuan et al. (2022) reported that DPPH and ABTS values recovered after in vitro digestion were 7% and 12%, respectively, and that there was no significant difference between HPP‐treated and untreated samples after the digestion process.

## 4 Conclusion

The present study showed that the hierarchy for bioactive component content and antioxidant capacity was Sarıcakaya‐1 > İzmir‐16 > Devedişi. HPH treatment resulted in an overall decrease in TSS and instrumental color values (L*, a*, and b*) and an increase in pH and *a*_*w*_ values of PJS. The overall color change (Δ*E*) was more visible at 100 MPa and 150 MPa HPH treatments. The effect of genotype and HPH treatment on the antimicrobial activity of PJS was insignificant. Compared with the Control groups, HPH processing increased the TPC, DPPH‐ARA, ABTS‐ARA, and FRAP values of freshly squeezed PJS up to 10.83%, 12.54%, 17.88%, and 21.20%, respectively. On the other hand, initial TAC and AAC of HPH‐treated PJS decreased by 5.66% and 18.57%, respectively. After in vitro gastrointestinal digestion, dramatic losses in TPC (66.90%–72.12%), TAC (98.47%–98.26%), and AAC (77.96%–84.68%) were observed compared with initial values, depending on genotype and treatment. In the same vein, DPPH‐ARA, ABTS‐ARA, and FRAP values recovered after intestinal digestion were 12.47%–18.40%, 23.97%–35.61%, and 18.72%–22.11%, respectively. HPH processing did not adversely affect the bioaccessibility of bioactive components and the recovered antioxidant capacity of freshly squeezed PJS. Presenting even positive effect, the 150 MPa HPH treatment of Saricakaya‐1 had the highest values for TPC bioaccessibility and recovered ABTS‐ARA after in vitro gastrointestinal digestion. Considering the overall data, the best results were obtained in Sarıcakaya‐1 genotype and 150 MPa HPH treatments. Future studies are needed to combine HPH processing with a controlled cooling system to prevent the increase of product outlet temperature and minimize the loss of heat‐sensitive components of PJS.

## Author Contributions

**Emre Turan:** formal analysis (equal), investigation (equal), methodology (equal), resources (equal), visualization (equal), writing – original draft (equal), writing – review and editing (equal). **Rafet Aslantaş:** conceptualization (equal), investigation (equal), methodology (equal). **Jale Bilgin:** formal analysis (equal), methodology (equal), resources (equal). **Muhammet Irfan Aksu:** conceptualization (equal), data curation (equal), investigation (equal), visualization (equal), writing – review and editing (equal).

## Conflicts of Interest

The authors declare no conflicts of interest.

## Supporting information


Table S1.

## Data Availability

The data that support the findings of this study are available from the corresponding author upon reasonable request.
