# Supplementary material for: High‐Pressure Homogenization of Pomegranate Juice: Impact on Physicochemical, Antioxidant, Antimicrobial, and In Vitro Bioaccessibility Properties
Source: Food Sci Nutr. 2024 Nov 7;12(12):10315–29. doi: 10.1002/fsn3.4571 (PMC11666902; doi:10.1002/fsn3.4571)
Supplement: Supplementary file 1 — Table S1. [file FSN3-12-10315-s001.docx]

**Title**

**High pressure homogenization as an innovative approach for minimally processed pomegranate juice from different genotypes: Changes in physicochemical properties, antimicrobial activity, *in vitro* bioaccessibility and antioxidant capacity**

Emre Turan^a^, Rafet Aslantaş^b^, Jale Bilgin^b^, Muhammet Irfan Aksu^c*^

*^a^ Ordu University, Faculty of Agriculture, Department of Food Engineering, 52200 Ordu, Türkiye*

*^b^ Eskişehir Osmangazi University, Faculty of Agriculture, Department of Horticulture, 26160, Eskişehir, Türkiye*

*^c^Atatürk University, Faculty of Agriculture, Department of Food Engineering, 25240, Erzurum, Türkiye*

*Corresponding Author

Atatürk University, Faculty of Agriculture, Department of Food Engineering, Erzurum, Türkiye

*E-mail*: [miaksu@atauni.edu.tr](mailto:miaksu@atauni.edu.tr); miaksu@hotmail.com (M.I. Aksu)

Tel: +90 (442) 2312428; Fax: +90 (442) 231 5878

**Table S1.** Some pomological characteristics of pomegranate genotypes used in the present study

| **Pomological Character** | **Genotypes** | | |
| --- | --- | --- | --- |
|  | **Devedişi** | **İzmir-16** | **Sarıcakaya-1** |
| Fruit weight (g) | 344.1 | 317.5 | 291.3 |
| Aril yield (%) | 62.6 | 57.2 | 56.1 |
| 100 aril weight (g) | 57.2 | 48.1 | 41.6 |
| Juice yield (%) | 53.2 | 49.3 | 48.0 |
| Aril color (Visually) | Light red | Red | Dark red |
